# Factors associated with door-in to door-out delays among ST-segment elevation myocardial infarction (STEMI) patients transferred for primary percutaneous coronary intervention: a population-based cohort study in Ontario, Canada

**DOI:** 10.1186/s12872-018-0940-z

**Published:** 2018-10-29

**Authors:** Oumin Shi, Anam M. Khan, Mohammad R. Rezai, Cynthia A. Jackevicius, Jafna Cox, Clare L. Atzema, Dennis T. Ko, Thérèse A. Stukel, Laurie J. Lambert, Madhu K. Natarajan, Zhi-jie Zheng, Jack V. Tu

**Affiliations:** 1 School of Public Health, Shanghai Jiaotong University School of Medicine, South Chongqing Road No, Shanghai, 227 China; 2 Institute for Clinical Evaluative Sciences, G1 06, 2075 Bayview Avenue, Toronto, ON Canada; 3 Western University of Health Sciences, 309 E 2nd St, Pomona, California, USA; 4 Schulich Heart Centre, Sunnybrook Health Sciences Centre, 2075 Bayview Avenue, Toronto, ON Canada; 5 University of Toronto, 27 King’s College Circle, Toronto, ON Canada; 6 Department of Medicine, Hamilton Health Sciences, McMaster University, 1200 Main St W, Hamilton, ON Canada; 7 Sunnybrook Health Sciences Centre, 2075 Bayview Avenue, Toronto, ON Canada; 8 Dalhousie University, 6299 South St, Halifax, NS Canada; 9 Cardiology Evaluation Unit, Institut national d’excellence en santé et en services sociaux (INESSS), 2021, Avenue Union, Bureau 10.083, Montréal, Québec Canada

**Keywords:** ST-segment elevation myocardial infarction (STEMI), Primary percutaneous coronary intervention (PCI), Door-in to door-out (DIDO), Pre-hospital electrocardiogram (ECG), Mortality

## Abstract

**Background:**

Compared to ST-segment elevation myocardial infarction (STEMI) patients who present at centres with catheterization facilities, those transferred for primary percutaneous coronary intervention (PCI) have substantially longer door-in to door-out (DIDO) times, where DIDO is defined as the time interval from arrival at a non-PCI hospital, to transfer to a PCI hospital. We aimed to identify potentially modifiable factors to improve DIDO times in Ontario, Canada and to assess the impact of DIDO times on 30-day mortality.

**Methods:**

A population-based, retrospective cohort study of 966 STEMI patients transferred for primary PCI in Ontario in 2012 was conducted. Baseline factors were examined across timely DIDO status. Multivariate logistic regression was used to examine independent predictors of timely DIDO as well as the association between DIDO times and 30-day mortality.

**Results:**

The median DIDO time was 55 min, with 20.1% of patients achieving the recommended DIDO benchmark of ≤30 min. Age (OR_> 75 vs 18–55_ 0.30, 95% CI: 0.16–0.56), symptom-to-first medical contact (FMC) time (OR_61-120mins vs < 60mins_ 0.60, 95% CI: 0.39–0.90; OR_>120mins vs < 60mins_ 0.53, 95% CI:0.35–0.81) and emergency medical services transport with a pre-hospital electrocardiogram (ECG) (OR_EMS transport + ECG vs self-transport_ 2.63, 95% CI:1.59–4.35) were the strongest predictors of timely DIDO. Patients with timely ECG were more likely to have recommended DIDO times (33.0% vs 12.3%; *P* < 0.001). A significantly higher proportion of those who met the DIDO benchmark had timely FMC-to-balloon times (78.7% vs 27.4%; *P* < 0.001). Compared to patients with DIDO time ≤ 30 min, those with DIDO times > 90 min had significantly higher adjusted 30-day mortality rates (OR 2.82, 95% CI:1.10–7.19).

**Conclusions:**

While benchmark DIDO times were still rarely achieved in the province, we identified several potentially modifiable factors in the STEMI system that might be targeted to improve DIDO times. Our findings that patients who received a pre-hospital ECG were still being transferred to non-PCI capable centres suggest strategies addressing this gap may improve patient outcomes.

**Electronic supplementary material:**

The online version of this article (10.1186/s12872-018-0940-z) contains supplementary material, which is available to authorized users.

## Background

For patients with ST-Elevation myocardial infarction (STEMI), time-to-treatment is an important modifiable determinant of survival [1, 2]. Timely primary percutaneous coronary intervention (PCI) is considered the preferred method of reperfusion compared to fibrinolytic therapy, given its lower STEMI mortality rate [3]. However, about 25% of Canadian residents do not live within a one-hour drive of a PCI-capable centre and often have to be transferred from a non-PCI capable (referral) hospital [4]. Consequent lengthening in receipt of PCI therapy has a demonstrably negative impact on clinical outcomes [5, 6].

Delays in providing timely reperfusion therapy can occur at several points along the treatment pathway but take place frequently at the referral hospital [7, 8]. Guidelines for STEMI recommend that the time between arrival to, and transfer from a referral hospital to a PCI-capable hospital, also referred to as the door-in-door-out (DIDO) time, should be ≤30 min, and has been widely adopted as an important metric for quality of STEMI care [1, 9]. However, several studies have noted that this benchmark is rarely achieved [7, 8, 10–12].

In light of this, there is a clear need to develop new strategies or refine pre-existing ones that are responsible for prolonged DIDO times. One such suggestion is having emergency medical services (EMS) personnel administer a 12-lead electrocardiogram (ECG) prior to arrival at the hospital (pre-hospital ECG) for patients suspected of having a STEMI, although the impact on reducing DIDO times has not been well studied [13].

Utilizing a population-based cohort of STEMI patients in Ontario, Canada, we aimed to identify potentially modifiable factors to improve DIDO time. A secondary objective was to examine the impact of DIDO times on 30-day mortality rates adjusted for important confounders. Our findings could have important public health and policy implications, providing ‘real world’ evidence for areas in the STEMI system that should be targeted for improvement.

## Methods

### Data sources

The Ontario portion of the Canadian Institute for Health Information (CIHI) Discharge Abstract Database (DAD) and National Ambulatory Care Reporting System (NACRS) were used to identify all patients who were hospitalized or presented to an emergency department (ED) for a STEMI event in calendar year 2012. Detailed information on patients was obtained from their medical charts which were abstracted by trained cardiology nurses hired by the research team. Abstracted data included patient characteristics at baseline, key time variables, presenting information and information on the patient transport process. The data were securely transmitted electronically to a database housed at the Institute for Clinical Evaluative Sciences (ICES) in Toronto, Ontario. Given the low-risk nature of this study, Ontario privacy laws allow waivers of informed consent to abstract the data [14, 15].

Mortality information was obtained through linkage, utilizing encoded health card numbers, to the Registered Persons Database (RPDB). The RPDB contains socio-demographic and date of death information on all Ontario residents eligible for the Ontario Health Insurance Plan.

### Study population

**Fig. 1:**
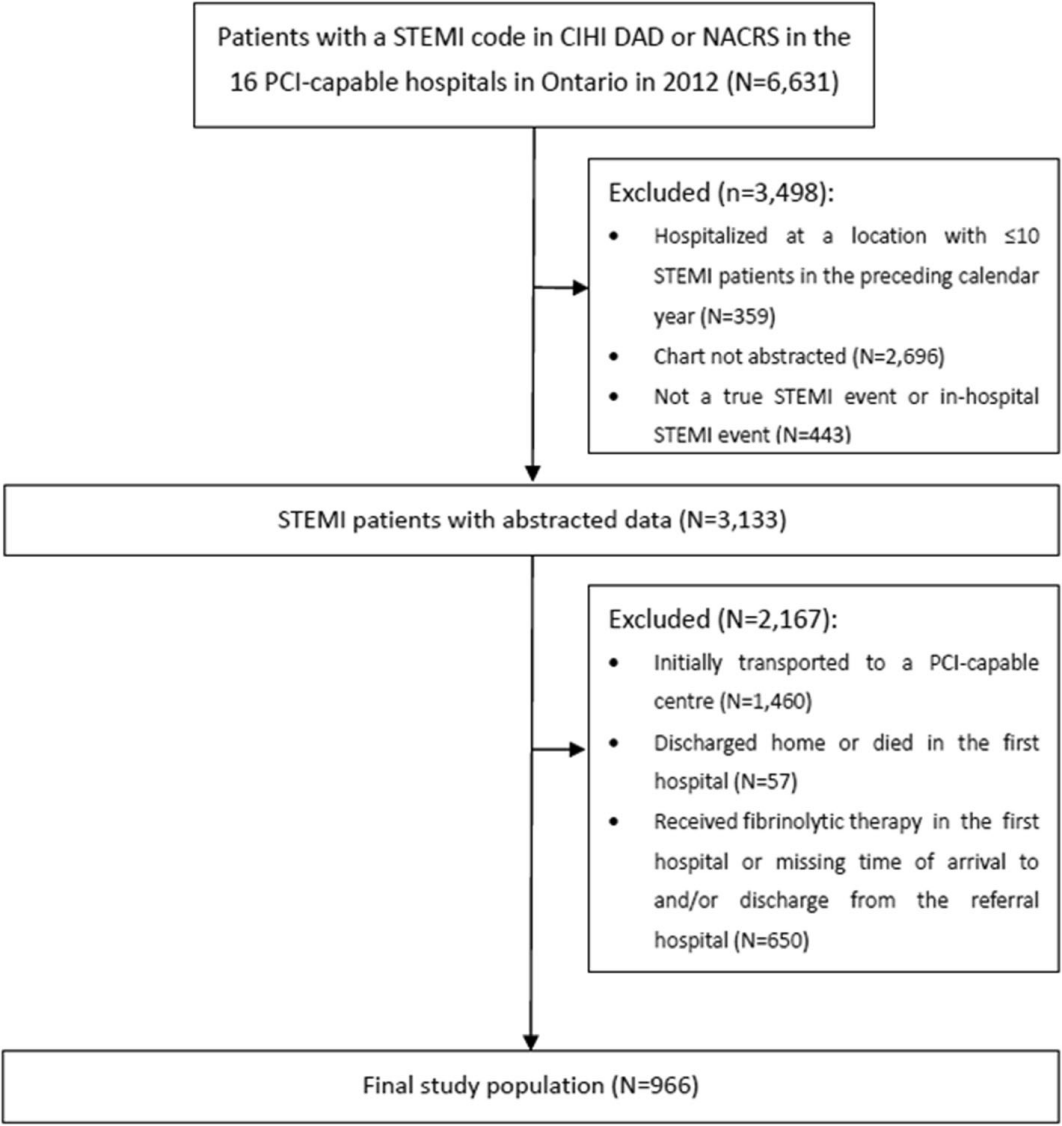
Cohort creation. CIHI, Canadian Institute for Health Information; DAD, Discharge Abstract Database; NACRS, National Ambulatory Care Reporting System; PCI, percutaneous coronary intervention; STEMI, ST-segment elevation myocardial infarction

Figure 1 details the creation of the study cohort. International Classification of Diseases, tenth revision codes (Table S1 in the Data Supplement [see Additional file 1]) were used to identify 6631 patients who had a recorded STEMI event in administrative data. A random sample of ~ 50% of the patients in each health region, termed a Local Health Integration Network (LHIN), had their charts abstracted, affording us a representative sample of patients for use in the study. Based on chart reviews, individuals who did not meet the clinical criteria for a STEMI event or were determined to have had an in-hospital STEMI were further excluded. Among 3133 STEMI patients with abstracted charts, we focused on the 1616 patients (51.6%) who initially presented to a referral hospital and thus excluded those who were transported directly to a PCI-capable centre or who were discharged home or died in the first hospital. For the purpose of this study, in which timeliness of DIDO for primary PCI was examined, we further excluded those who received fibrinolytic therapy at the first hospital or were missing information on the variables required to compute DIDO time, creating the final study population of 966 patients.

### Process of care and clinical outcome definitions

We assessed several important process of care measures along the STEMI treatment pathway. Where appropriate, benchmarks were selected to be consistent with published Canadian and American guidelines for STEMI care [1, 9, 13, 16]. Details on the operationalization of the measures and their associated benchmarks can be found in Table S2 of the Data Supplement [see Additional file 1].

Our primary clinical outcome of interest was all-cause 30-day mortality, defined as death due to any cause within 30 days of the primary PCI procedure.

### Statistical analyses

Baseline patient and process of care measures were examined across timely DIDO status. The proportion of those achieving timely DIDO who also met the benchmark first medical contact (FMC)-to-balloon time of ≤120 min was also examined.

The median durations of process of care indicators comprising the total symptom-to-reperfusion time were compared amongst those who were transported by EMS but did not receive a pre-hospital ECG, transported by EMS and received a pre-hospital ECG and those who transported themselves to hospital.

Generalized estimating equation (GEE) multivariate logistic regression was used to compute odds ratios and corresponding 95% confidence intervals (CI) to identify baseline characteristics independently associated with a DIDO time ≤ 30 min [17]. GEE models were used to account for clustering at the LHIN level given that patients residing in the same LHIN may be similarly affected by health system factors which effect STEMI care (e.g., regional STEMI networks) [13]. A similar regression model was used to assess the association between DIDO times and 30-day mortality adjusted for patient factors including demographics, traditional cardiac risk factors and co-morbidities, presenting features, symptom-to-FMC time, pre-hospital ECG status and the median durations of several process-of-care measures.

For comparative purposes, we also calculated the crude 30-day mortality rates amongst the 518 patients who received fibrinolytic therapy at the referral hospital and had data on the times of arrival to the hospital and administration of therapy. This group was not the major focus of this study and as noted earlier, was excluded in the cohort used for the main set of analyses.

Where there was missing data for a variable, it was < 1% of the sample size and was imputed to the most common value.

All analyses were conducted at ICES in Toronto, Canada using SAS version 9.3 (SAS Institute, Cary NC). This study was approved by the Research Ethics Boards at Sunnybrook Health Sciences Centre and each of the PCI centres in Ontario.

## Results

### Patient characteristics and timely DIDO

The distribution of DIDO times is shown in Figure S1 in the Data Supplement [see Additional file 1]. The median DIDO time was 55 min (interquartile range: 35–112 min). Only 194 patients (20.1%) achieved the DIDO benchmark, and ~ 1/3 of patients had DIDO times greater than 90 min.

Fewer patients with DIDO times ≤30 min, compared to patients with times > 30 min, were elderly, defined as older than 75 years of age (10.3% vs 19.9%; adjusted OR [aOR] _> 75 vs 18–55_ 0.30, 95% CI: 0.16–0.56) (Table 1). Traditional cardiovascular risk factors, co-morbidities and presenting information were not found to be significantly associated with DIDO timeliness.

**Table 1:** Baseline characteristics of the study cohort across Door-in to door-out (DIDO) status, Ontario, Canada, 2012

	DIDO time (mins)		aOR (95% CI)^a^ (timely DIDO vs. untimely)^b^
	≤30 min	> 30 min	
	(*N* = 194)	(*N* = 772)	
	Frequency (column %)		
Age group, years			
18–55	87 (44.8)	230 (29.8)	Ref.
56–65	51 (26.3)	234 (30.3)	0.57 (0.39–0.87)
66–75	36 (18.6)	154 (19.9)	0.61 (0.37–0.99)
> 75	20 (10.3)	154 (19.9)	0.30 (0.16–0.56)
Sex, females	33 (17.0)	194 (25.1)	0.72 (0.46–1.15)
Cardiovascular risk factors			
Diabetes mellitus	39 (20.1)	167 (21.6)	0.95 (0.62–1.45)
Current smoker	88 (45.4)	302 (39.1)	0.96 (0.67–1.39)
Hypertension	93 (47.9)	390 (50.5)	0.98 (0.68–1.41)
Previous cardiovascular clinical events			
Myocardial infarction	21 (10.8)	99 (12.8)	0.68 (0.70–3.03)
Angina	8 (4.1)	41 (5.3)	0.93 (0.39–2.22)
COPD	7 (3.6)	38 (4.9)	0.94 (0.38–2.33)
Stroke	7 (3.6)	30 (3.9)	1.43 (0.58–3.57)
Presenting characteristics			
Cardiac arrest at scene	13 (6.7)	51 (6.6)	0.69 (0.71–2.04)
Elevated cardiac enzymes^c^	171 (88.1)	675 (87.4)	1.22 (0.71–2.04)
Off-hours presentation^d^	122 (62.9)	508 (65.8)	0.91 (0.64–1.28)
Symptom to FMC time, mins			
0–60	84 (43.3)	212 (27.5)	Ref.
61–120	56 (28.9)	268 (34.7)	0.60 (0.39–0.90)
> 120	54 (27.8)	292 (37.8)	0.53 (0.35–0.81)
Transport to first hospital			
Self-transport	103 (53.1)	510 (66.1)	Ref.
EMS transport with ECG	36 (18.6)	71 (9.2)	2.63 (1.59–4.35)
EMS transport without ECG	55 (28.4)	191 (24.7)	1.45 (0.95–2.22)

*Abbreviations*: *aOR* adjusted odds ratio, *CI* confidence interval, *COPD* chronic obstructive pulmonary disease, *DIDO* door-in to door-out, *ECG* electrocardiogram, *EMS* emergency medical services, *FMC* first medical contact, *mins* minutes, *Ref* reference

^a^Logistic regression model fully adjusted for all the variables in the table

^b^Door-in to door-out times were considered timely if they were ≤ 30 min

^c^Elevated cardiac enzyme levels were defined as having at least one of the following occur within the first 24 h of the first medical contact: 1) a rise in troponin levels above the upper reference limit or the level indicative of acute myocardial infarction, or 2) a rise in creatine kinase MB or creatine kinase more than twice the upper limit of normal as defined on the lab report

^d^Defined as presentation to a hospital before 9 am or after 5 pm on weekdays and anytime on weekends

Approximately 43% of patients with timely DIDO had acceptable symptom-to- FMC contact times of between 0 and 60 min, in contrast to only 27.5% of those with untimely DIDO (Table 1). We observed a strong gradient for timeliness of DIDO, such that those who had the longest symptom-to-FMC times had an almost 2-fold decrease in timely DIDO rates compared to those with the shortest time (aOR_61-120mins vs. 0–60 min_ 0.60, 95% CI: 0.39–0.90; aOR_>120mins vs. 0–60 min_ 0.53, 95% CI: 0.35–0.81) (Table 1).

### Process of care measures

**Fig. 2:**
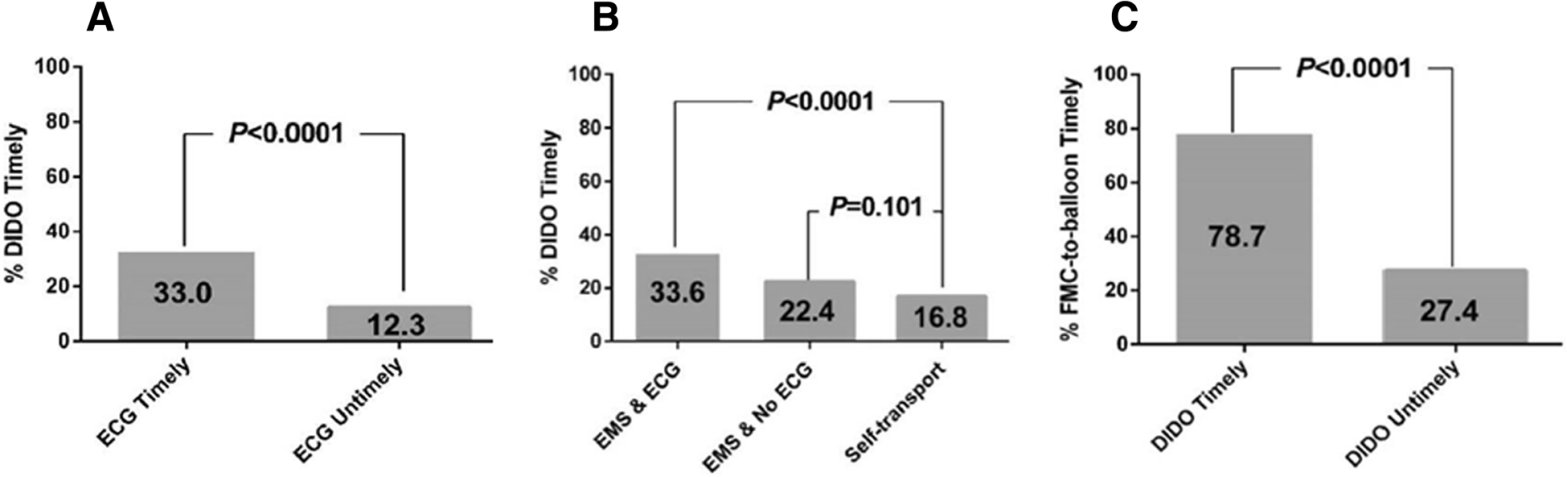
Prevalence of door-in to door-out times of ≤30 min (timely) across timely electrocardiogram (≤10 min) status (**a**), and hospital transport groups (**b**). Percentage of patients who achieved the first medical contact-to-balloon benchmark of ≤120 min across timely door-in to door-out status (**c**). DIDO, door-in to door-out; ECG, electrocardiogram; EMS, emergency medical services; FMC, first medical contact

As depicted in Fig. 2a, receipt of a timely ECG (time to first ECG was less than 10 min) was significantly associated with timely DIDO (*P* < 0.0001). Amongst process of care measures, EMS transport with receipt of a pre-hospital ECG was the strongest independent predictor of timely DIDO (aOR_EMS + pre-hospital ECG vs self-transport_ 2.63, 95% CI: 1.59–4.35) (Table 1; Fig. 2b).

### Timely reperfusion

A significantly (*P* < 0.0001) higher proportion of those who met the DIDO benchmark also had timely FMC-to-balloon times, with rates almost three times higher in the former group (78.7% vs 27.4%) (Fig. 2c). Overall, 47.4% of the transferred patients in our study met the FMC-to-balloon time benchmark.

**Fig. 3:**
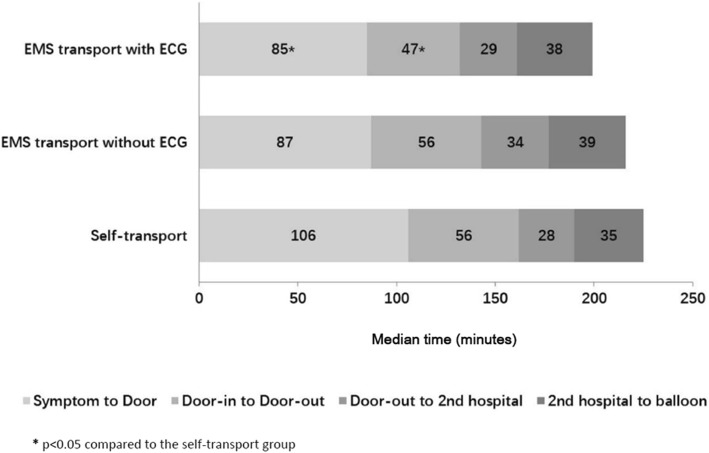
Components of median time to reperfusion across hospital transport groups

The breakdown of the components of median reperfusion time across those who self-transported to hospital, were transported by EMS but did not receive a pre-hospital ECG, and those who were transported by EMS and had a pre-hospital ECG are shown in Fig. 3. The median times were 225, 216 and 199 min, respectively. Symptom-to-door time accounted for the greatest duration of median reperfusion time, followed by DIDO times, which were significantly shorter amongst those who were transported by EMS and received pre-hospital ECG compared to the self-transport group (Symptom-to-door time: 85 min vs 106 min, DIDO: 47 min vs 56 min; *P* < 0.05). 33.6% of those in the former group had timely DIDO compared to only 22.4% and 16.8% in the EMS and no pre-hospital ECG group and self-transport groups, respectively (Fig. 2B).

### 30-day mortality

Table 2 shows 30-day mortality rates across DIDO times. Crude 30-day mortality rates ranged from 4.1% amongst those with DIDO times of ≤30 min to 11.6% in those with DIDO times in excess of 90 min. While only patients with DIDO times > 90 min had significantly higher mortality rates (aOR 2.82, 95% CI: 1.10–7.19) a mortality gradient was evident.

**Table 2:** Association between door-in to door-out times and 30-day all-cause mortality amongst patients transferred for primary percutaneous coronary intervention, Ontario, Canada, 2012

	Number of events / Patient population	Crude 30-day mortality rate (%)	aOR (95% CI)^a^
DIDO time (mins)			
≤ 30 (timely)	8/194	4.1	Ref.
31–60	17/ 333	5.1	1.05 (0.38–2.89)
61–90	12/145	8.3	1.73 (0.58–5.09)
> 90	34/294	11.6	2.82 (1.10–7.19)
Overall	71/966	7.3	N/A

*Abbreviations*: *aOR* adjusted odds ratios, *CI* confidence intervals, *DIDO* door-in to door-out, *mins* minutes, *N/A* not applicable, *Ref* reference

^a^Logistic regression model was adjusted for patient demographics, traditional cardiac risk factors and co-morbidities, presenting features, symptom-to-FMC time, pre-hospital ECG status and times for process-of-care measures

Patients who received fibrinolytic therapy in the first hospital were excluded from the main analyses, but their crude 30-day mortality rate (6.6%) did not differ significantly from patients transferred for primary PCI (7.3%; *P* = 0.676). Crude mortality rates for those administered fibrinolytic therapy can be found in Table S3 in the Data Supplement [see Additional file 1]. Patients with DIDO times > 90 min had significantly higher crude mortality rates than those who got timely fibrinolytic therapy (11.6% vs. 5.5%*; P* = 0.012).

## Discussion

In the present study, we utilized a population-based cohort of STEMI patients in Ontario, Canada to identify potentially modifiable factors that can improve DIDO times and the impact of DIDO times on long-term mortality. Only 20% of STEMI patients who were transferred from a referral hospital to a PCI-capable centre for primary PCI met the DIDO time of ≤30 min. Age was the strongest patient-level predictor of DIDO times. Transport to hospital by EMS in combination with receiving a pre-hospital ECG was the strongest process of care measure associated with DIDO times. In particular, this group had substantively shorter symptom to reperfusion times compared to those who transported themselves to hospital, driven largely by significantly shorter symptom to door and DIDO times. The mortality gradient for DIDO times observed in our study is important as it demonstrates poor long-term outcomes for those who experienced longer DIDO times. The crude 30-day mortality rates in patients transferred for primary PCI was higher than those who got fibrinolytic therapy, in particular for those with DIDO times over 90 min.

Our finding that achievement of benchmark DIDO time was low (20%) is consistent with previous studies [7, 8, 10–12]. A study using national registry data from the United States reported that only 11% of patients achieved DIDO benchmarks, and other studies reported rates of ~ 10% [8, 10, 11]. Canadian studies have reported higher rates than American-based studies, one reported 14% in Quebec [7, 10]. Several factors could account for the disparities in rates including varying characteristics of the study population, regional differences in policies and design of the STEMI system and/or changes in trends over time [7, 8, 10–12]. The latter may be especially pertinent in this case given that our work is more recent than other cited studies.

The guidelines indicate that 90% of patients transferred from a referring hospital should meet the 120-min time-to-treatment standard [1]. Less than 50% of transferred patients in our study met the FMC-to-balloon time benchmark. We observed stark differences in achievement of FMC-to-balloon time benchmark across DIDO status. Most transferred patients with DIDO time > 30 min do not have absolute contraindications for fibrinolytic therapy [18, 19]. In our and previous studies, about half of transferred patients had a DIDO time > 60 min, with few of these individuals receiving timely PCI [7, 8, 10–12]. Delayed transfer of STEMI patients with DIDO time > 60 min may not be a superior strategy to the timely use of fibrinolytic therapy [1, 3, 20]. One potential option to address prolonged DIDO times might be to increase the use of fibrinolytic therapy at referral hospitals, followed by transfer for catheterization post-lytic therapy [21].

Elderly age was found to be one of the strongest predictors of DIDO time in our study which is in light with previous studies [7, 10]. Elderly individuals may be less likely to present with classic STEMI symptoms (e.g., retrosternal chest pain) and/or more likely to have ECGs which are complex to interpret, that may delay diagnosis and recognition of a STEMI. Our findings highlight there is a need to bring awareness to the complexity and non-traditional nature of the symptoms with which these elderly patients sometimes present in order to improve DIDO times for this group. Prior studies have shown the disparities in time to reperfusion for STEMI patients in the elderly [22, 23]. Better software that can accurately read difficult ECGs, enhanced training for medical personnel and public awareness campaigns might improve DIDO times.

We observed that patients transported by EMS personnel, whether in association with a pre-hospital ECG or not, resulted in shorter symptom-to-door times compared to those self-transported patients. However, DIDO times were found to be significantly shorter compared to patients who self-transported only amongst those who received a pre-hospital ECG, suggesting that while EMS transport, irrespective of pre-hospital ECG status affects symptom-to-door time, for DIDO times it is the receipt of pre-hospital ECG that makes a difference. Provision of a pre-hospital ECG was not a universally adopted or mandated practice in Ontario in 2012 [9]. However, the findings of the present study suggest that policies which mandate and fund equipping ambulances with this technology and training EMS personnel to administer these ECG’s might have a significant impact on reduction of DIDO times and mortality.

Ideally, provision of a pre-hospital ECG should allow for a quicker diagnosis, thus facilitating the activation of STEMI protocols, result in more direct transfers to PCI-capable centres or mitigate delays in the referral ED [24]. Notably, our study found that a fair proportion (11.1%) of individuals received a pre-hospital ECG and were still initially transferred to a non-PCI capable hospital. Contributing factors may include diagnostic uncertainty, incorrect interpretation of the ECG and/or a clinically unstable patient. It is also plausible that some ambulances continue to bring patients to the nearest community hospital, regardless of ECG findings because the PCI-capable centre is further away, which would identify an area to target for improvement. Increased clarity in the guidelines around when EMS personnel should bypass the closest centre in favor of a more distant PCI-capable hospital, which is integrated within a STEMI protocol, may in part help alleviate this problem. In 2015, a STEMI bypass protocol in Ontario enables paramedics to bypass local hospitals and transport patients with STEMI directly to PCI-capable centres if arrival at the PCI centre will be ≤60 min from the first medical contact [9]. These guidelines came into effect in the province in 2017 and future studies should examine whether new guidelines have reduced the number of patients with STEMI-positive ECG’s being transferred to non-PCI capable hospitals. Our findings show that the major delay in reperfusion times occurs at the referral hospital, in line with previous studies’ findings [10, 25]. However, it should also be recognized that it may be difficult in EDs to provide care within guideline recommendations for every patient, especially for those with atypical presentations.

The present study aids in generalizability of findings to other populations. The fact that we examined important process of care measures including receipt of pre-hospital ECG’s, which have not been extensively studied enabled us to assess the impact of current STEMI management recommendations and guidelines on DIDO times so that we can ascertain a more complete picture of the STEMI care system and identify areas for improvement. Additionally, the present study found DIDO times to be an independent predictor of 30-day mortality, with the observed gradient strengthening the conclusion, and suggests the adverse impact of long DIDO times extends beyond the hospital visit itself. Previous studies have observed similar gradients for in-hospital mortality only or have been underpowered to make inferences regarding longer-term mortality outcomes adjusted for important confounders [10].

However, the present study presents with some important limitations. First, information on key time intervals was ascertained through retrospective chart review and recorded times could not be independently validated. Secondly, we were unable to collect information on the distance to the referral hospital or between the referral hospital and the PCI-capable centre or the details of patient symptoms as they were not available or well documented. Lastly, the administrative STEMI codes we used have not been validated. However, they were applied consistently across all records and those who were identified as STEMI cases from administrative data had their diagnosis verified via chart review by a trained cardiology research nurse, making it unlikely that non-STEMI cases were included in the study.

## Conclusions

In conclusion, our findings suggest that benchmark DIDO times were not achieved for a majority of transferred STEMI patients in the province. Patient age and process of care measures, namely symptom-to-FMC time and receipt of a pre-hospital ECG, were identified to be strong independent predictors of DIDO time. Despite provision of pre-hospital ECGs, a fair proportion of patients were still transferred to non-PCI capable hospitals, suggesting that policies and system-level changes aimed at bypassing non-PCI capable hospitals in favor of PCI-capable hospitals for those with STEMI positive ECGs could have an important impact on outcomes.

## Additional file

Additional file 1: Data supplement containing additional tables and figures (e.g., disease classification codes, benchmark times etc.). (DOCX 130 kb)

## Abbreviations

AOR: Adjusted odds ratio
CI: Confidence interval
CIHI: Canadian Institute for Health Information
COPD: Chronic obstructive pulmonary disease
D2B: Door-to-balloon time
DAD: Discharge Abstract Database
DIDO: Door-in to door-out
ECG: Electrocardiogram
EMS: Emergency medical services
FMC: First medical contact
mins: Minutes
NACRS: National Ambulatory Care Reporting System
PCI: Percutaneous coronary intervention
Ref: Reference
STEMI: ST-segment elevation myocardial infarction
TRANSFER-AMI: Trial of Routine Angioplasty and Stenting after Fibrinolysis to Enhance Reperfusion in Acute Myocardial Infarction
