# Supplementary material for: Factors associated with door-in to door-out delays among ST-segment elevation myocardial infarction (STEMI) patients transferred for primary percutaneous coronary intervention: a population-based cohort study in Ontario, Canada
Source: BMC Cardiovasc Disord. 2018 Oct 29;18:204. doi: 10.1186/s12872-018-0940-z (PMC6206901; doi:10.1186/s12872-018-0940-z)
Supplement: Supplementary file 1 — Data supplement containing additional tables and figures (e.g., disease classification codes, benchmark times etc.). (DOCX 130 kb) [file 12872_2018_940_MOESM1_ESM.docx]

**DATA SUPPLEMENT**

**Factors associated with door-in to door-out delays among ST-segment elevation myocardial infarction (STEMI) patients transferred for primary percutaneous coronary intervention: a population-based cohort study in Ontario, Canada**

**Contents:**

**Table 1** International Classification of Diseases (ICD-10) ST-segment elevation myocardial infarction (STEMI) codes.

**Table 2** ST-segment elevation myocardial infarction (STEMI) process of care measures and associated benchmarks.

**Figure 1** Distribution of door-in to door-out (DIDO) times (minutes)

**Table 3** Crude 30-day mortality rate for ST-segment elevation myocardial infarction (STEMI) patients with abstracted data but who received fibrinolytic therapy in the first hospital

**Table S1** International Classification of Diseases (ICD) ST-segment elevation myocardial infarction (STEMI) codes

| **ICD-10 code** | **Description** | **Main or secondary diagnosis** |
| --- | --- | --- |
| I210 | Acute transmural myocardial infarction of anterior wall | Main |
| 1211 | Acute transmural myocardial infarction of inferior wall | Main |
| I212 | Acute transmural myocardial infarction of other sites | Main |
|  |  |  |
| I213 | Acute transmural myocardial infarction of unspecified site | Main |
| I214 | Acute subendocardial myocardial infarction | Main |
|  |  |  |
| I219 | Acute myocardial infarction, unspecified | Main |
| I220 | Subsequent myocardial infarction of anterior wall | Main |
| I221 | Subsequent myocardial infarction of inferior wall | Main |
| I228 | Subsequent myocardial infarction of other sites | Main |
| I229 | Subsequent myocardial infarction of unspecified site | Main |
| I240 | Coronary thrombosis not resulting in myocardial infarction | Main |
| R9430 | Electrocardiogram suggestive of ST segment elevation myocardial infarction [STEMI] | Secondary |
|  |  |  |

ICD, International Classification of Diseases.

**Table S2** ST-segment elevation myocardial infarction (STEMI) process of care measures and associated benchmarks

| **Process of care measures** | **Definition** | **Benchmark Applied** |
| --- | --- | --- |
| Door-in to door-out (DIDO) time | Elapsed time between arrival at, and discharge from a STEMI referral hospital | ≤ 30 minutes |
| First medical contact-to-balloon time | Duration of time between first medical contact and receipt of PCI | ≤ 120 minutes from the first medical contact for patients transferred from a referral hospital |
| Receipt of a timely ECG | ECG administered by EMS (pre-hospital ECG) for those arriving by ambulance or upon arrival at hospital for those transporting themselves | ≤ 10 minutes |
| Symptom-to-first medical contact time | Elapsed time from symptom onset to being attended to by paramedics for those transported by EMS, or nurses and/or doctors after arrival at the ED for those who transported themselves to hospital | NONE |
| Symptom-to-door time | Length of time between symptom onset and arrival at the first hospital | NONE |
| Door-out to arrival at the second hospital time | Time from discharge at the first, referral hospital, to arrival at the second hospital | NONE |
| Second hospital to balloon time | Duration of time from arrival at the second, PCI-capable hospital, to receipt of PCI | NONE |

ECG, electrocardiogram; ED, emergency department; EMS, emergency medical services; PCI, percutaneous coronary intervention; STEMI, ST-segment elevation myocardial infarction.

**References**

1. O'Gara PT, Kushner FG, Ascheim DD, Casey DE, Chung MK, de Lemos JA*, et al*. 2013 ACCF/AHA Guideline for the Management of ST-Elevation Myocardial Infarction: A Report of the American College of Cardiology Foundation/American Heart Association Task Force on Practice Guidelines. Circulation. 2013; 127(4):e362-e425.
2. CCN. Cardiac Care Network of Ontario. Ontario STEMI Bypass Protocol. 2015.

**
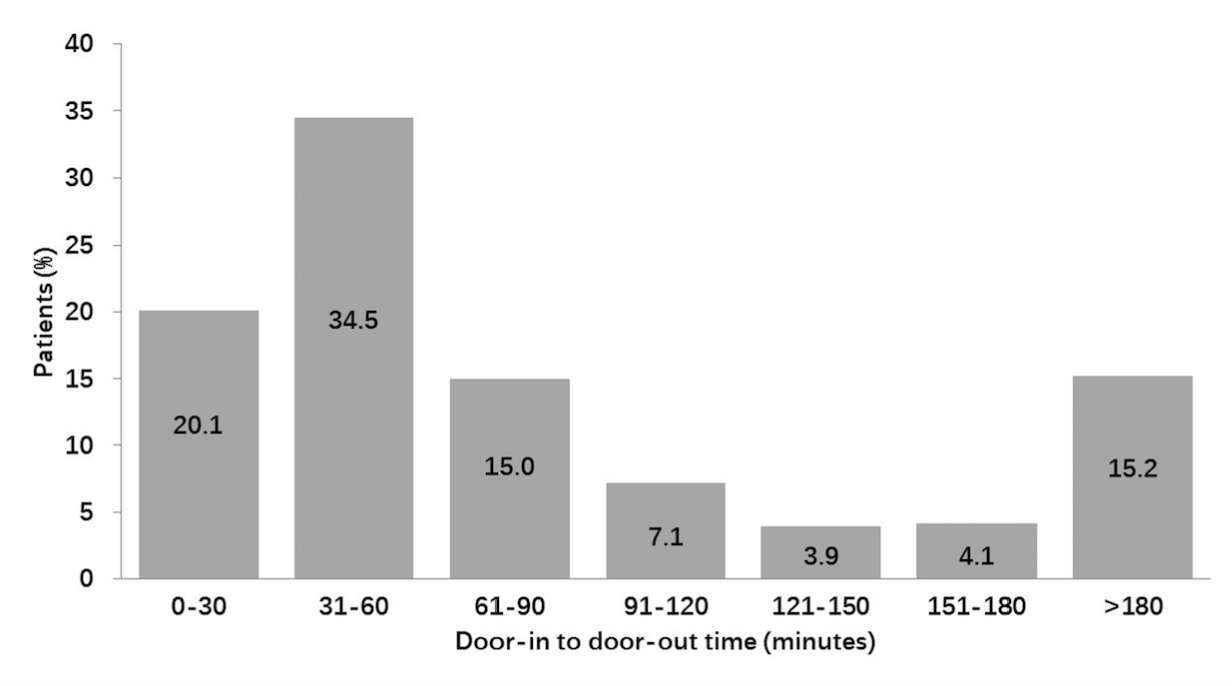
**

**Fig. S1** Distribution of door-in to door-out times, Ontario, Canada, 2012

**Table S3** Crude 30-day mortality rate for ST-segment elevation myocardial infarction (STEMI) patients with abstracted data but who received fibrinolytic therapy in the first hospital, Ontario, Canada, 2012

|  | Number of events/  Patient population | Crude 30-day mortality rate (%) |
| --- | --- | --- |
| **Overall** | 34/518 | 6.6 |
| Timely | 14/256 | 5.5 |
| Untimely | 20/262 | 7.6 |

Analysis was conducted amongst a sub-set of ST-segment elevation myocardial infarction (STEMI) patients in our study who had their charts abstracted but who were administered fibrinolytic therapy at the first hospital and as such were excluded from the final cohort used in the study used to study door-in to door-out times.
